# Supplementary material for: Seminoma and Embryonal Carcinoma Footprints Identified by Analysis of Integrated Genome-Wide Epigenetic and Expression Profiles of Germ Cell Cancer Cell Lines
Source: PLoS One. 2014 Jun 2;9(6):e98330. doi: 10.1371/journal.pone.0098330 (PMC4041891; doi:10.1371/journal.pone.0098330)
Supplement: File S1 — ZIP file containing DMRforPairs output for significant regions. Please start from the html files. (ZIP) [file pone.0098330.s008.zip › figures/27621.pdf]

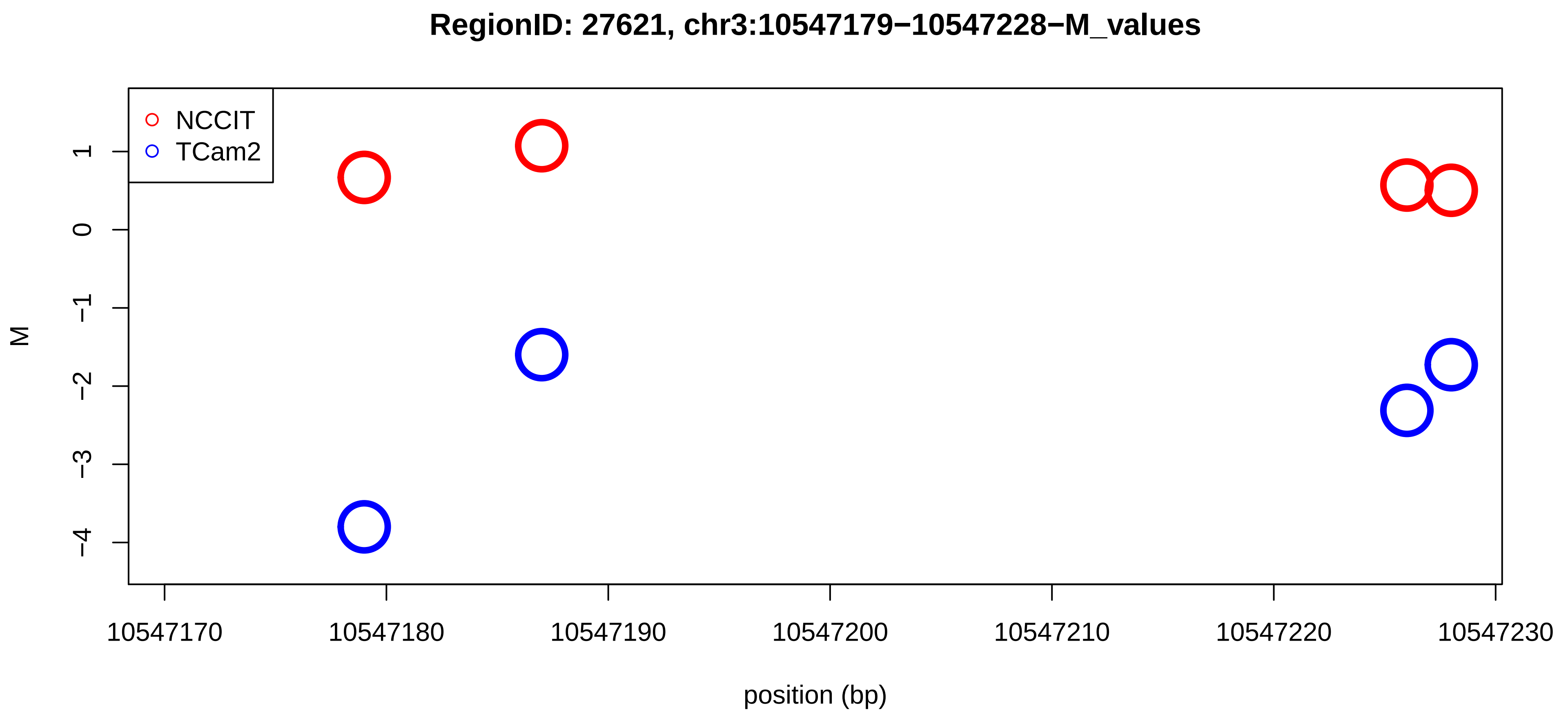

RegionID: 27621, chr3:10547179−10547228−M_values
NCCIT
TCam2
M
1
0
−1
−2
−3
−4
10547170
10547180
10547190
10547200
10547210
10547220
10547230
position (bp)

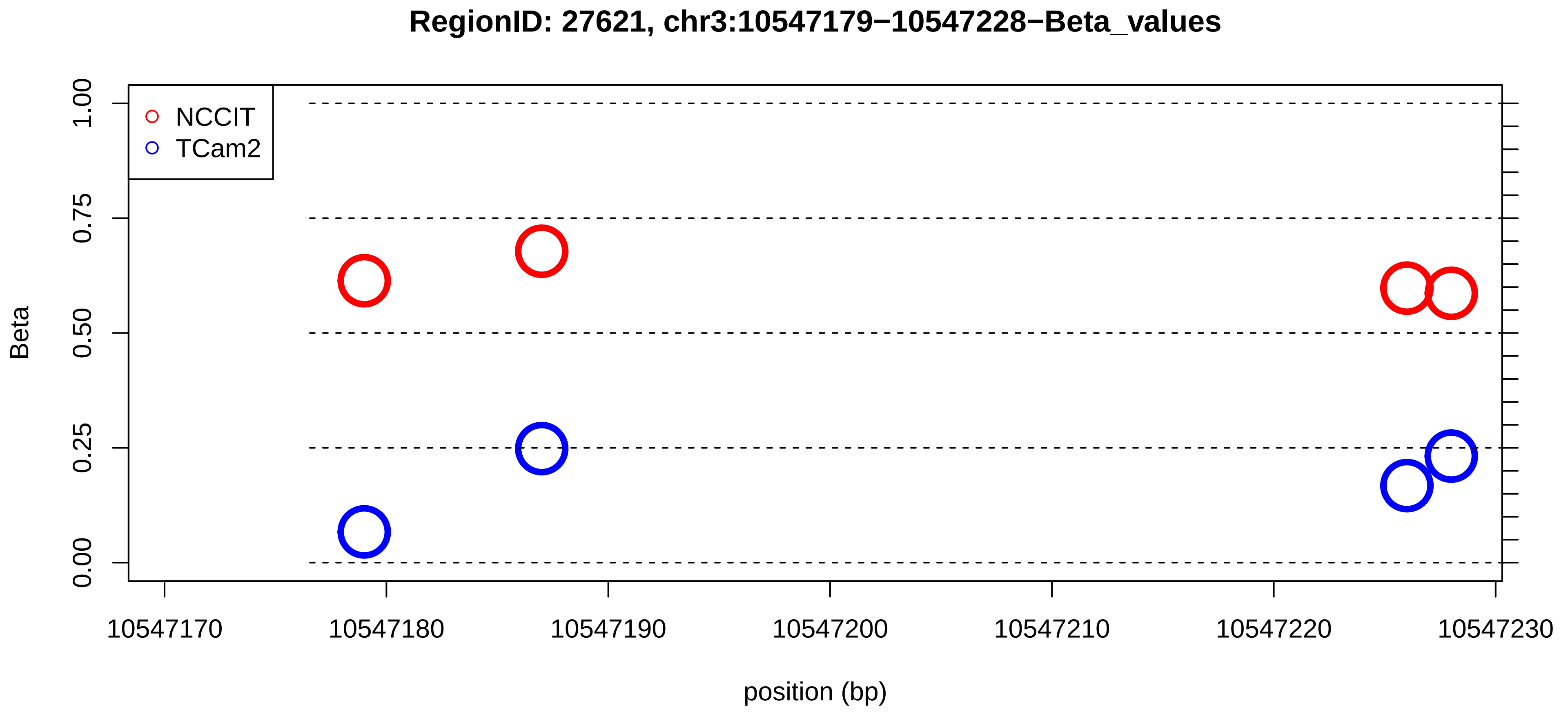

RegionID: 27621, chr3:10547179−10547228−Beta_values
NCCIT
TCam2
Beta
1.00
0.75
0.50
0.25
0.00
10547170
10547180
10547190
10547200
10547210
10547220
10547230
position (bp)
